# MEFV gene mutation distrubution in Azerbaijan population

**DOI:** 10.1186/1546-0096-13-S1-P127

**Published:** 2015-09-28

**Authors:** A Berdeli, G Mukhtarova, A Oz, S Musayev

**Affiliations:** 1 Ege University Medical Faculty, Pediatric Department and Moleculer Medicine, Izmir, Azerbaijan; 2 Azerbaijan Medical University. Baku, Azerbaijan

## Introduction

Familial Mediterranean fever (FMF)(MIM 249100) is a hereditary autoinflammatory disorder characterized by episodes of inflammation in the absence of high-titer autoantibodies or antigen-specific T cells. The Mediterranean fever (MEFV) gene(OMIM 608107) located on chromosome 16p13.3, which encodes the 781-amino-acid protein pyrin, is the causative gene for this monogenic Mendelian disease. This study presents the molecular analysis of an MEFV gene mutation screen of 268 patients from Azerbaijan Republic, with clinical diagnoses of FMF.

## Materials and methods

Genomic DNA was obtained from peripheral blood. 4 exons of MEFV gene were analysed by PCR and direct DNA sequencing method. Furthermore the obtained nucleotid sequence compaired with reference sequence published in NCBI (NM_000243.2).

## Results

%56 of 268 patients were found to have mutations. Allele frequency of common five major mutation of MEFV gene E148Q,M680I, M694V, V726A and R761H were 8.2%;1.3%;,3.1%, 9.8%; and %4.2 respectively. it is revealed one novel MEFV mutation E148D in 3 patients wich updated to INFEVERS. The mutation carrier frequency of majör common alleles was 15% in healthy individuals from Azerbaijan population. In addition, the frequency of R202Q polymorphism in patients group was 45%. and 32% in control group in this population.

## Conclusion

The mutation carrier frequency and disease causative common major MEFV gene mutations in Azerbaijan population are similar of certain ethnic groups. Moreover the mutation of R761H more frequently, compared to other ethnic groups, may be a founder effect.

